# Chemokine-Armed Oncolytic Viruses: Engineering Immune Cell Trafficking to Transform the Tumor Microenvironment

**DOI:** 10.3390/pharmaceutics18080950

**Published:** 2026-07-31

**Authors:** Akram Alwithenani

**Affiliations:** Department of Clinical Laboratory Sciences, College of Applied Medical Sciences, Umm Al-Qura University, Makkah, Saudi Arabia; aiwithenani@uqu.edu.sa

**Keywords:** oncolytic virus, chemokine, immune cell trafficking, tumor microenvironment, CXCL11, CCL5, cold tumor, immunotherapy

## Abstract

Most patients with solid tumors do not respond to immune checkpoint blockade, and inadequate T cell infiltration of the tumor parenchyma is the dominant mechanism of primary resistance. Oncolytic viruses address this problem by a distinct route: they replicate selectively within tumor cells, produce immunogenic cell death, and convert infected cells into local sources of any encoded transgene. Most armed designs to date have carried cytokine or checkpoint-antibody payloads, and chemokines have attracted comparatively little attention despite bearing directly on the trafficking bottleneck. This review synthesizes the preclinical literature on chemokine-armed oncolytic viruses across three receptor axes: CXCR3 (CXCL9, CXCL10, CXCL11), CCR5 (CCL5/RANTES), and CCR7 (CCL19). The accumulated evidence indicates that therapeutic outcome depends less on the chemokine payload itself than on the interaction between payload and viral backbone. CXCL11 outperforms its sister CXCR3 ligands not through intrinsic potency but because it is non-redundant with the endogenous chemokines induced by vesicular stomatitis virus and vaccinia, and because it largely escapes proteolytic cleavage by dipeptidyl peptidase 4 (DPP4). CCL5 has shown the most consistent activity in dual-payload designs that pair chemotaxis with a T cell survival cytokine such as IL-15. CCL19, which addresses lymphoid organization rather than effector recruitment, rests on a single published construct. One evidence gap is central: no head-to-head comparison of chemokine payloads within a single viral platform has been published. We therefore propose a translational decision framework that aligns chemokine selection with the immune contexture of the target tumor.

## 1. Introduction

Immune checkpoint inhibitors have established that endogenous antitumor immunity, once relieved of inhibitory regulation, can mediate durable cancer regression [1,2]. Most patients with solid tumors nonetheless fail to respond to checkpoint blockade as monotherapy, and inadequate T cell infiltration of the tumor parenchyma is the principal mechanism of primary resistance [1,2,3,4]. In the cancer-immunity cycle described by Chen and Mellman, chemokine-mediated trafficking occupies a non-redundant position: without a functional gradient to guide primed T cells from the draining lymph node to the tumor bed, upstream priming cannot translate into effector activity [4,5,6]. Tumors dismantle this gradient by several parallel mechanisms. PRC2/EZH2-mediated H3K27 methylation of the CXCL9 and CXCL10 promoters silences their expression [7,8]. The atypical chemokine receptor ACKR3/CXCR7, frequently overexpressed in tumors, internalizes chemokine ligands without signaling and depletes the gradient before it can establish itself [9]. FAP^+^ carcinoma-associated fibroblasts secrete CXCL12 in quantities sufficient to make the stroma a chemokine barrier that excludes effector T cells [6,10,11].

**Figure 1:**
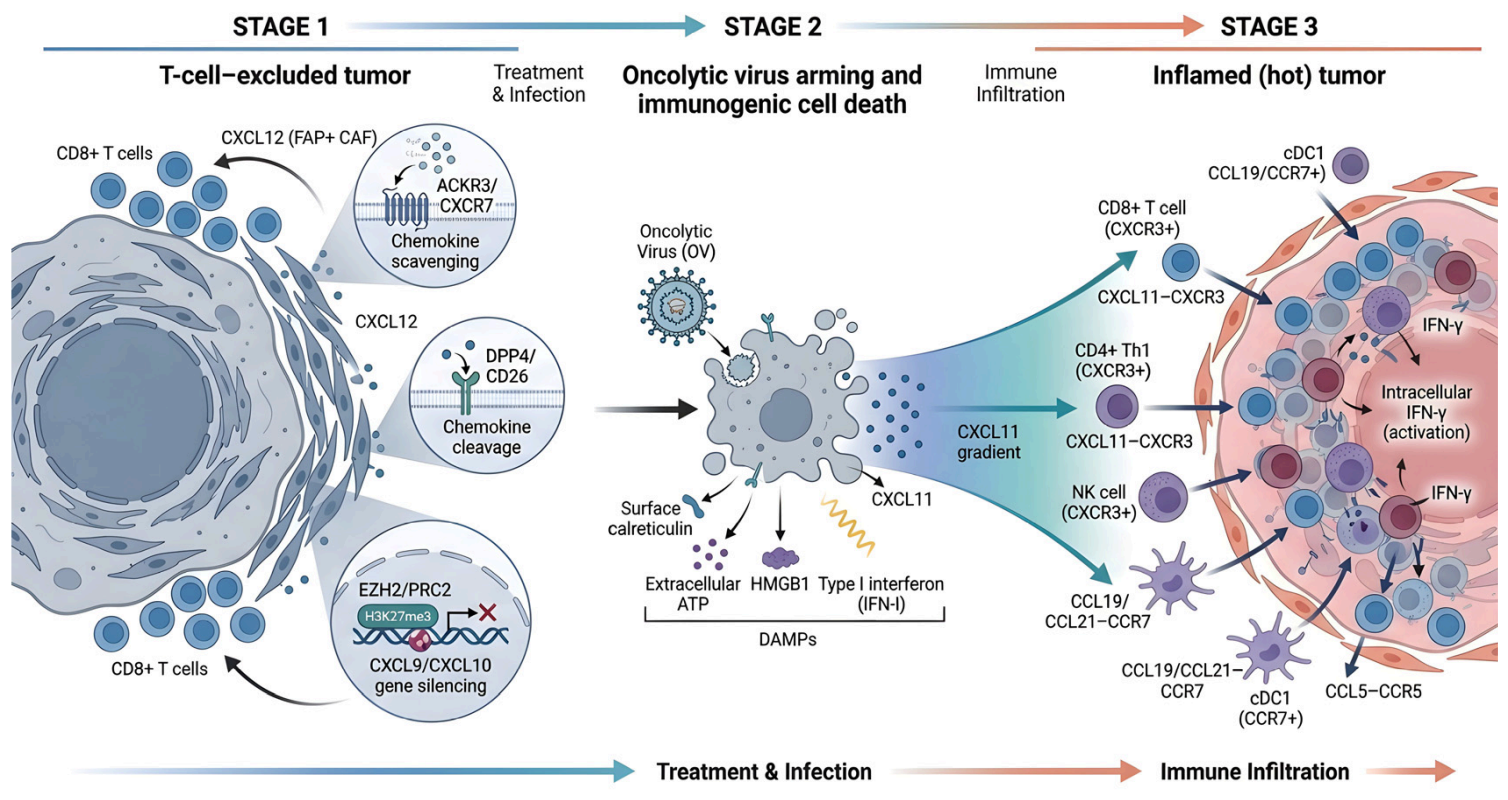
**Chemokine-armed oncolytic viruses convert immunologically “cold” tumors to “hot.”** (**Left**) In T cell–excluded tumors, effector T cells are retained at the invasive margin by stromal barriers: CXCL12 secreted by FAP^+^ cancer-associated fibroblasts, chemokine scavenging and N-terminal cleavage by ACKR3/CXCR7 and DPP4/CD26, and EZH2/PRC2-mediated H3K27me3 silencing of the CXCL9 and CXCL10 promoters. (**Center**) Intratumoral oncolytic virus infection triggers immunogenic cell death with release of DAMPs (surface-exposed calreticulin, ATP, HMGB1, and type I interferon), while a virus-encoded CXCL11 transgene is secreted to form a local chemokine gradient. (**Right**) CXCL11–CXCR3, CCL5–CCR5, and CCL19/CCL21–CCR7 signaling recruits CD8^+^ effector and Th1 CD4^+^ T cells, NK cells, and cDC1 dendritic cells into the tumor core, generating an inflamed, IFN-γ–rich “hot” microenvironment. Created in BioRender. Alwithenani, A. (2026) https://BioRender.com/24vgfpx (accessed on 28 July 2026).

Among the modalities of modern cancer immunotherapy, which include checkpoint inhibitors, adoptive cell therapies, cancer vaccines, and cytokine therapies, oncolytic viruses occupy a distinct niche: they lyse tumor cells directly and remodel the immune microenvironment in situ [3,12,13]. That dual action makes them well suited to the trafficking bottleneck [3,12,13,14,15]. Unlike systemically administered small molecules or antibodies, which must access the tumor through the same vasculature that excludes T cells, oncolytic viruses replicate selectively within tumor cells and convert infected cells into self-amplifying, spatially restricted sources of transgene expression [3,16]. Armed oncolytic virus therapy is already clinically viable: talimogene laherparepvec (T-VEC), an HSV-1 armed with GM-CSF, was approved for advanced melanoma in 2015 [17], and teserpaturev (G47Δ) received conditional approval in Japan for recurrent glioblastoma in 2021 [18]. Ribas and colleagues subsequently showed that intratumoral T-VEC increased CD8^+^ T cell density in both injected and non-injected melanoma lesions before pembrolizumab dosing, direct clinical evidence that oncolytic viruses can convert immune-cold tumors to immune-hot states [1] (Figure 1).

Unmodified oncolytic viruses do not, however, generate chemokine environments that are predictable or optimized for recruitment of specific effector populations [19,20,21]. Viral backbones induce distinct innate immune signatures: vaccinia virus drives robust CXCL10 and CCL5 induction, whereas vesicular stomatitis virus produces a more interferon-dominated profile with variable chemokine output [19,20,22]. That variability is the rationale for engineering oncolytic viruses with defined chemokine transgenes, an approach that allows controlled, therapeutically optimized gradients to be superimposed on the endogenous viral response [23,24,25,26]. Chemokine arming is therefore mechanistically distinct from cytokine arming. Cytokine cargoes such as IL-12, IL-15, and GM-CSF act on effector function once T cells reach the tumor, whereas chemokine cargoes determine whether they arrive at all [14,27,28].

The oncolytic viruses in clinical and preclinical development span several backbones with distinct biology. Herpes simplex virus type 1 (HSV-1) is the most clinically advanced platform, since both T-VEC and G47Δ are HSV-1, and its large transgene capacity suits it well to arming [17,18]. Vaccinia virus combines rapid cytoplasmic replication, systemic spread, and a large payload capacity, and is a frequent chassis for chemokine arming [23,24,29]. Adenoviruses, including conditionally replicating designs, are the most widely used platform in preclinical chemokine studies and support high transgene expression [25,30,31]. Vesicular stomatitis virus, Newcastle disease virus, reovirus, and measles virus complete the panel under active investigation, each with a characteristic innate-immune and chemokine-induction profile [19,20,22,32]. Across these platforms, oncolytic viruses share a common role in modern immunotherapy: they act as in situ vaccines that convert immunologically cold tumors into inflamed lesions responsive to checkpoint blockade [1,3,17].

These advantages are tempered by well-recognized limitations. Pre-existing and treatment-induced neutralizing antibodies, together with rapid hepatic and splenic sequestration, constrain systemic delivery and largely confine current agents to intratumoral administration of accessible lesions [3,16]. Antiviral innate responses can curtail replication and transgene expression before a therapeutic gradient is established, and the balance between antiviral and antitumor immunity is difficult to predict across patients [19,20,22]. Several strategies address these barriers: cell-based carrier delivery and polymer or liposomal shielding to evade neutralization, backbone and capsid engineering to modulate tropism and innate sensing, and, most consequentially for this review, transgene arming to steer the induced response toward durable antitumor immunity, particularly in combination with immune checkpoint inhibitors [1,3,13,14]. Insufficient and poorly controlled effector-cell trafficking is one such challenge, and it is the specific problem chemokine arming is designed to solve.

This review synthesizes the preclinical literature on chemokine-armed oncolytic viruses and identifies the priorities for clinical translation. Section 2 provides a brief primer on the chemokine receptor axes most relevant to antitumor immunity. Section 3 through Section 5 examine the published constructs grouped by target receptor axis: CXCR3 (Section 3), CCR5 and CCR7 (Section 4), and the chemokines that recruit dendritic cells (Section 5). Section 6 and Section 7 take up two underexamined dimensions of the field: the influence of viral backbone selection on the outcome of chemokine arming, and the design space opened by multi-payload constructs. Section 8 surveys the antagonism mechanisms tumors deploy against chemokine signaling and considers their relevance to virus-delivered chemokines. Section 9 closes with a translational decision framework pairing chemokine selection with tumor immune contexture [2,5,33,34], the principal evidence gaps, and recommendations for biomarker-guided clinical trial design.

## 2. Chemokine Biology Primer: Receptor Axes Relevant to Antitumor Immunity

The chemokine superfamily comprises approximately fifty ligands acting through twenty receptors to govern immune cell migration under both homeostatic and inflammatory conditions [26]. A defined subset of these ligand–receptor axes bears directly on the recruitment of effector lymphocytes, dendritic cells, and natural killer cells into the tumor microenvironment [26,35,36]. Rational chemokine payload selection in oncolytic virus design depends on how these axes are regulated.

### 2.1. The CXCR3 Axis: CXCL9, CXCL10, and CXCL11

CXCR3 is the principal receptor mediating effector CD8^+^ T cell and Th1 CD4^+^ T cell migration into inflamed tissues and tumors, and it is also expressed on a subset of natural killer cells, placing it at the center of the trafficking step that limits antitumor immunity [26,35,36]. It binds three interferon-γ-inducible ligands, CXCL9 (MIG), CXCL10 (IP-10), and CXCL11 (I-TAC), with a defined affinity hierarchy in which CXCL11 binds most tightly and CXCL9 most weakly [26,35]. The three ligands share a receptor but are not functionally interchangeable. Differences in affinity, in engagement of the atypical receptor ACKR3/CXCR7, and in susceptibility to proteolytic processing give them distinct pharmacological behavior, which is central to payload selection in oncolytic virus design. The axis has accordingly been proposed both as a biomarker of T-cell-inflamed tumors and as a therapeutic target [37,38,39]. CXCL9 and CXCL10 are produced predominantly by macrophages and dendritic cells in response to IFN-γ, generating a feed-forward loop in which an initial wave of T cell infiltration amplifies further recruitment. A chemokine-armed virus must either ignite that circuit or substitute for it in a tumor where it has been silenced [37,40]. All three CXCR3 ligands are subject to post-translational regulation by dipeptidyl peptidase 4 (DPP4/CD26), and for CXCL11 by CD13/aminopeptidase N acting in concert with DPP4, which remove N-terminal residues and convert the products from receptor agonists into antagonists that bind and desensitize CXCR3 without initiating signaling [41,42,43,44]. CXCL10 is the most susceptible to this inactivation, whereas CXCL11 retains comparatively greater resistance. That distinction, together with the affinity hierarchy, makes CXCL11 the most rational CXCR3 payload and recurs throughout the constructs discussed below [41,42,45].

The CXCR3 ligands also carry prognostic and predictive weight in clinical oncology. High intratumoral expression of CXCL9, CXCL10, and CXCL11 correlates with denser CD8^+^ T cell infiltration and more favorable outcomes across multiple solid tumor types, including colorectal, ovarian, breast, and melanoma cohorts [37,38,39]. The same ligands recur in the T-cell-inflamed gene-expression signatures used to stratify immunotherapy benefit. The IFN-γ-related mRNA profile of Ayers and colleagues, which predicted response to pembrolizumab across melanoma, head and neck, and gastric cancer, includes CXCL9, CXCL10, and CXCL11 among its core genes [38]. The expanded pan-tumor analysis of Cristescu and colleagues confirmed that this signature predicts response to PD-1 blockade, both as monotherapy and within combination chemo-immunotherapy regimens, independently of tumor mutational burden [46]. Tumors with low CXCR3-ligand expression are characteristically immune-excluded and respond poorly, which is why the axis is now read as a marker of the inflamed phenotype rather than of tumor burden [37,39]. Prognostic association in untreated tumors and predictive value for immunotherapy response together provide the epidemiological rationale for restoring CXCR3-ligand gradients therapeutically.

That rationale has been tested directly, and the resulting therapeutic literature is the strongest argument for using the CXCR3 ligands as payloads. Epigenetic silencing of CXCL9 and CXCL10 by EZH2 and DNMT1 restrains effector T cell infiltration in ovarian cancer, and pharmacological inhibition of these enzymes restores chemokine expression, increases T cell homing, and improves the efficacy of PD-L1 blockade and adoptive cell therapy [7]. Inhibiting DPP4 with sitagliptin preserves the full-length agonist form of CXCL10, enhances lymphocyte trafficking, and augments both spontaneous tumor immunity and checkpoint blockade in preclinical models [41]. Chemokine-receptor engineering of adoptive cell products pursues the same trafficking objective from the receptor side rather than the ligand side [47,48]. Each of these strategies acts systemically or requires ex vivo manipulation of the cell product. An oncolytic virus offers a complementary option: delivery of the ligand itself, at the tumor site, under the control of viral replication. That is the rationale developed in the sections that follow.

### 2.2. The CCR5 Axis: CCL5/RANTES

CCR5 is expressed on activated T cells, NK cells, and monocytes, and is engaged by CCL3 (MIP-1α), CCL4 (MIP-1β), and CCL5 (RANTES) [26,49]. Of these ligands, CCL5 has attracted the most attention in oncolytic virus engineering, owing to its broad chemoattractant activity and established role in T cell tumor homing [25,49,50,51]. CCL5 and the CXCR3 ligands cooperate in a two-stage geometry: CCL5 supplies the longer-range signal that draws effector T cells toward the tumor margin, and CXCL9 directs final positioning within the parenchyma [40]. This division of labor has been shown in ovarian and colorectal cancer models, and it is the rationale for dual-axis strategies that may recruit more effectively than single-axis approaches [40,49].

The clinical literature on CCL5 is more equivocal than that on the CXCR3 ligands, and the difference bears directly on its use as a payload. High CCL5 expression is associated with favorable outcomes where it accompanies effector T cell and NK cell infiltration, but also with tumor progression, metastasis, and poor prognosis in breast, cervical, and gastric cancer, settings in which the same gradient recruits regulatory T cells and myeloid-derived suppressor cells and sustains a tumor-promoting stroma [52]. The therapeutic corollary has been tested in the opposite direction: CCR5 blockade with maraviroc repolarized tumor-associated macrophages and produced objective responses in patients with treatment-refractory colorectal cancer liver metastases [49]. Virally restricted intratumoral delivery is therefore best understood as an attempt to capture the recruitment benefit while avoiding the sustained systemic exposure implicated in the tumor-promoting phenotype.

### 2.3. The CCR7 Axis: CCL19 and CCL21

CCR7 is classically associated with homeostatic lymphocyte trafficking, where its ligands CCL19 and CCL21 organize naïve T cells and mature dendritic cells within the T cell zones of secondary lymphoid organs [53]. In tumors, CCR7 signaling has been linked to the formation of tertiary lymphoid structures (TLS), which themselves correlate with improved immunotherapy responses [54,55]. Arming an oncolytic virus with CCL19 therefore pursues a different objective from CXCR3- or CCR5-targeted strategies: not direct effector T cell recruitment, but local organization of antigen presentation and priming of antitumor responses within the tumor itself [29,53,55]. This objective rests on outcome data as well as mechanism: mature tertiary lymphoid structures and high intratumoral CCL19/CCL21 expression correlate with denser T and B cell infiltration and with longer survival, and in melanoma the presence of TLS-associated B cell signatures predicted response to checkpoint blockade independently of CD8^+^ T cell density [56]. CCR7-axis arming is therefore directed at a prognostically validated tissue structure rather than at cell recruitment alone.

### 2.4. Emerging Axes: CCR2, CX3CR1, and DC-Recruiting Chemokines

The CCR2/CCL2 axis plays a dual role in tumor immunology. CCL2 recruits inflammatory monocytes that may differentiate into tumoricidal macrophages, but it also draws in myeloid-derived suppressor cells and tumor-associated macrophages of the immunosuppressive M2 phenotype [57,58]. CX3CL1 (fractalkine), acting through CX3CR1, recruits both cytotoxic T cells and natural killer cells, and intratumoral expression has been associated with improved prognosis in gastric and colorectal cancers [59]. Dendritic cell recruitment depends on a different set of ligands: the CXCR3 ligands acting on conventional type 1 dendritic cells (cDC1s), together with the natural killer cell-derived chemokines XCL1 and CCL5 that recruit Batf3-dependent cDC1s into the tumor microenvironment [60,61,62,63].

## 3. The CXCR3 Axis: Lessons from CXCL9, CXCL10, and CXCL11-Armed OVs

The CXCR3 axis is the most extensively investigated chemokine target in oncolytic virus engineering, with published constructs spanning all three ligands across multiple viral platforms [19,23,24,30,31,64,65]. The accumulated evidence reveals substantial differences in therapeutic efficacy among the three ligands, and receptor binding affinity alone does not account for them. They reflect an interplay between chemokine biology, viral immunology, and tumor-mediated antagonism. The preclinical chemokine-armed constructs discussed across Section 3, Section 4 and Section 5 are compiled in Table 1, which juxtaposes each construct’s payload, viral platform, tumor model, immune effects, and principal insight, and provides the empirical basis for the comparisons that follow.

### 3.1. VSV-CXCL9: A Cautionary Tale of the Ceiling Effect

Eckert and colleagues engineered vesicular stomatitis virus expressing CXCL9 (VSV-CXCL9) and evaluated it in syngeneic murine tumor models (Table 1) [19]. Although CXCL9 production by infected tumor cells was confirmed, VSV-CXCL9 conferred no survival advantage over the unarmed parental VSV-Δ51 [19]. The negative result is mechanistically informative. Vesicular stomatitis virus is a potent inducer of type I interferon, and that response drives high-level endogenous CXCL9 and CXCL10 production by infected and bystander cells. Against an already-saturated baseline, a transgenic CXCL9 cassette adds a signal the tumor microenvironment cannot distinguish from the chemokine the infection has already elicited [19,20,22]. This is a ceiling effect: the benefit of arming depends not on the absolute amount of chemokine a construct produces, but on the increment, it adds over what the viral backbone induces on its own. Payload selection therefore becomes a question of non-redundancy, and the principle recurs throughout this review. An exogenous chemokine yields measurable benefit only where the chosen platform leaves a corresponding deficit for it to fill [19,20,69]. The same CXCL9 payload that is redundant in an interferon-dominated VSV backbone might prove informative in a platform that induces little endogenous CXCR3 ligand, which underscores that the construct, not the chemokine alone, is the meaningful unit of comparison.

### 3.2. CXCL10-Based Approaches: The DPP4 Vulnerability

In colorectal cancer, CXCL10 has been raised pharmacologically rather than delivered as a viral transgene: Francis and colleagues showed that a chemokine-modulating cocktail increased endogenous CXCL10 and CCL5 while reducing the Treg-attracting chemokines CCL22 and CXCL12 in an MC38 colon carcinoma model, enhancing CD8^+^ T cell and NK cell trafficking when administered sequentially after an oncolytic poxvirus [65]. The limitation of this payload is shown most directly by a head-to-head comparison of the three CXCR3 ligands: Matsumoto and colleagues stably expressed CXCL9, CXCL10, or CXCL11 in a murine squamous cell carcinoma line and found that CXCL9- and CXCL11-expressing tumors were markedly growth-inhibited whereas CXCL10-expressing tumors were not, a difference attributed to the DPP4 cleavage site in the CXCL10 N-terminus together with DPP4 expression in the tumor stroma [45]. That dissociation, trafficking achievable but tumor control not, points to the constraint that dominates this payload: susceptibility to proteolytic inactivation. DPP4, expressed on stromal fibroblasts, tumor endothelial cells, and infiltrating immune cells, cleaves the N-terminal dipeptide of CXCL10 to generate a truncated form that occupies CXCR3 without activating it, functioning as a dominant-negative antagonist [41,42,43,45]. Barreira da Silva and colleagues demonstrated that DPP4 inhibition preserves bioactive CXCL10, restores T cell trafficking to tumors, and improves immunotherapy responses in multiple preclinical models [41]. These findings indicate that CXCL10-armed oncolytic viruses may require co-administration of a DPP4 inhibitor such as sitagliptin to achieve optimal activity, a pharmacological dependency that can be avoided by choosing a DPP4-resistant payload [41,44,45].

### 3.3. CXCL11: The Preferred CXCR3 Ligand for OV Arming

CXCL11 has shown more consistent efficacy than its sister CXCR3 ligands across multiple oncolytic virus platforms, and the reasons illuminate the broader logic of payload selection. As the highest-affinity CXCR3 ligand, CXCL11 combines potent effector recruitment with relative resistance to the DPP4-mediated inactivation that cripples CXCL10, and it is typically induced only modestly by common viral backbones, leaving room for a transgene to make a measurable difference [23]. CXCL11 is also the only CXCR3 ligand to engage the atypical receptor ACKR3/CXCR7; because ACKR3/CXCR7 scavenges and degrades CXCL11 without initiating signaling, this engagement is a potential liability rather than a benefit, and CXCL11 is preferred because its high CXCR3 affinity and resistance to proteolytic inactivation outweigh the scavenging, not because of its CXCR7 binding. Liu and colleagues showed that vaccinia virus expressing CXCL11 (vvDD-CXCL11) substantially increased intratumoral CD8^+^ cytotoxic T lymphocyte (CTL) numbers and granzyme B expression in murine mesothelioma (Table 1), producing both local tumor control and a systemic vaccination effect that protected against tumor rechallenge [23]. Abscopal immunity from a locally administered virus is mechanistically significant: CXCL11-driven recruitment does more than relocate pre-existing effectors into the lesion and instead establishes the conditions for cross-priming of tumor-specific T cells within the infected microenvironment, so that a locally seeded gradient gives rise to a systemic, antigen-specific response [23,70].

A delivery paradox reported by Moon and colleagues adds a further constraint. Intratumoral CXCL11 delivered via vaccinia virus enhanced adoptive T cell therapy and vaccine efficacy (Table 1), whereas CXCL11 secreted by genetically engineered T cells failed to improve outcomes [64]. The most plausible explanation is autocrine and paracrine CXCR3 desensitization: when migrating cells are themselves the chemokine source, the spatial gradient required for chemotaxis is abolished [36,64]. More recently, Wang and colleagues constructed an oncolytic adenovirus armed with CXCL11 (oAd-CXCL11) and demonstrated that it reprogrammed the immunosuppressive glioblastoma tumor microenvironment, increasing CD8^+^ T cells and M1-polarized macrophages while decreasing myeloid-derived suppressor cells, regulatory T cells, and M2 macrophages (Table 1) [30]. In combination with B7H3-targeted CAR-T cells, oAd-CXCL11 produced durable responses in models in which CAR-T monotherapy was ineffective [30].

### 3.4. Sequential and Combination CXCR3-Targeted Strategies

Francis and colleagues developed a chemokine modulation cocktail (CKM) of IFN-α, poly-I:C, and the COX-2 inhibitor celecoxib, showing that broad remodeling of the CXCR3 chemokine landscape can overcome immune exclusion in colorectal carcinoma models [65]. Sequential administration of vvDD-CXCL11 followed by the CKM cocktail produced synergistic effects, indicating that oncolytic virus-delivered chemokines can prime the tumor microenvironment for subsequent immunomodulatory interventions [23,65]. In a separate strategy, Fang and colleagues showed that an oncolytic adenovirus co-expressing CXCL9 and IL-15 produced potent antitumor activity in prostate cancer models, increasing both CD8^+^ T cell and total CD45^+^CD3^+^ infiltration and enhancing CAR-T cell accumulation and efficacy in vivo (Table 1) [31]. This construct is worth reading against the VSV-CXCL9 result above: the same payload that proved redundant in an interferon-dominated backbone became productive in an adenoviral vector inducing little endogenous CXCR3 ligand, and when paired with a cytokine that sustains the cells it recruits. The comparison isolates backbone context, rather than the chemokine itself, as the variable that determined outcome.

## 4. The CCR5 and CCR7 Axes: CCL5 and CCL19-Armed OVs

### 4.1. CCL5/RANTES-Armed Constructs

CCL5 is the most extensively validated chemokine in dual-payload oncolytic virus configurations and has shown consistent efficacy when paired with a T cell survival cytokine [25,66,67,68]. Nishio and colleagues established the paradigm. Their construct, Ad5Δ24 armed with both RANTES and IL-15, enhanced CAR-T cell migration to neuroblastoma xenografts (Table 1) and prolonged CAR-T persistence through IL-15-mediated survival signaling [25,66]. The design’s contribution was to address two distinct barriers, trafficking and persistence, within a single viral construct [25,28,66].

This dual-payload approach has been extended to additional tumor types. Fang and colleagues paired CA9-targeted CAR-T cells with an oncolytic adenovirus carrying CCL5 and IL-12 (Ad5-ZD55-hCCL5-hIL12) in renal cell carcinoma, demonstrating enhanced CAR-T infiltration accompanied by Stat4 activation in the tumor, and prolonged survival (Table 1) [67]. The Stat4 signal is mechanistically useful because it confirms that the IL-12 arm was acting through its canonical pathway in situ rather than merely being expressed, indicating that the two payloads engaged distinct and complementary mechanisms rather than converging on the same one. The same group subsequently developed OncoViron (AdKi67-C3), the most complex chemokine-armed oncolytic virus reported to date, encoding CCL5, IL-12, and IFN-γ under Ki67 promoter control (Table 1) [68]. Intratumoral OncoViron administration combined with B7H3-specific CAR-T cells produced complete tumor regression in xenograft models, eradicated non-injected distant tumors, and established long-term antitumor memory [68].

Two factors likely account for the consistent success of CCL5-based constructs. First, CCR5 expression on a broad range of activated immune effector cells produces a wide recruitment profile. Second, CCL5 cooperates with the CXCR3 ligands in establishing the hierarchical chemokine gradients described above [40,49,50]. Li and colleagues separately showed that vaccinia virus expressing CCL5 amplified endogenous antitumor immunity in murine colon carcinoma, increasing intratumoral T cell infiltration and enhancing the efficacy of subsequent vaccination strategies (Table 1) [24]. Notably, this single-payload construct improved the performance of a subsequent intervention rather than producing tumor regression on its own—a pattern consistent with recruitment achieved without an accompanying survival signal, and the clearest illustration in the tabulated record of why CCL5 has been more successful as one half of a dual-payload design than as a sole transgene. CCL5-armed oncolytic viruses may therefore function not solely as T cell chemoattractants but as immune adjuvants that reshape the tumor microenvironment more broadly.

### 4.2. CCL19-Armed OVs: An Underexplored Paradigm

Thorne and colleagues armed vaccinia virus with CCL19 (vvCCL19) and observed an unusual immunological profile: enhanced antitumor efficacy with increased immune cell infiltration (Table 1), but without the accelerated viral clearance that typically accompanies immune-stimulating modifications of oncolytic viruses [29]. One plausible interpretation is that CCR7-mediated recruitment preferentially attracts naïve and central memory T cells rather than the effector populations responsible for antiviral immunity, producing a more favorable ratio of tumor-reactive to virus-reactive T cells [29,53]. The adoptive cell therapy literature offers additional support: Adachi and colleagues showed that CAR-T cells engineered to co-express IL-7 and CCL19 substantially improved tumor infiltration and survival in solid tumor models [55]. vvCCL19 nonetheless remains the only published oncolytic virus armed with CCL19, an underexplored opportunity in the field [29].

## 5. Dendritic Cell-Recruiting Strategies

Conventional type 1 dendritic cells (cDC1s) are essential for cross-presentation of tumor antigens and initiation of de novo CD8^+^ T cell responses [60,61,62,63]. Spranger and colleagues showed that melanomas lacking cDC1 infiltration failed to recruit effector T cells regardless of checkpoint blockade, establishing cDC1 presence as a prerequisite for successful immunotherapy [60]. Böttcher and colleagues subsequently identified NK cell-derived CCL5 and XCL1 as the primary chemoattractants driving cDC1 migration into tumors and showed that this axis is actively suppressed by tumor-derived prostaglandin E2 [61]. Dangaj and colleagues defined the cooperative mechanism between constitutive (CCL5) and inducible (CXCL9) chemokines that enables T cell engraftment within tumors [40]. Arming oncolytic viruses with cDC1-recruiting chemokines such as XCL1 or CCL5 therefore addresses a bottleneck upstream of T cell infiltration, with the potential to convert immune desert tumors into sites capable of autonomous antigen presentation [60,61,63]. Huang and colleagues constructed a recombinant Newcastle disease virus expressing MIP-3α (NDV-MIP-3α) that recruited dendritic cells and induced immunogenic cell death in vitro and in murine melanoma, enhancing antitumor immunity (Table 1) [32]. The coupling is the significant feature: the same construct supplies both the chemotactic signal that brings dendritic cells to the lesion and the immunogenic cell death that gives them antigen to acquire once they arrive, addressing recruitment and antigen availability in a single agent. NDV-MIP-3α nonetheless remains the only published oncolytic virus armed with a dendritic cell-recruiting chemokine, leaving the XCL1 and CCL5 strategies outlined above untested in this configuration.

Viewed together, the preclinical constructs compiled in Table 1 reveal several cross-cutting patterns. First, single-chemokine arming succeeds or fails according to the redundancy between the transgene and the backbone’s endogenous output: CXCL9 delivered by VSV yields no survival benefit because the platform already saturates that ligand, whereas CXCL11, weakly induced by most backbones and resistant to DPP4 cleavage, produces tumor regression and even abscopal immunity [19,23]. Second, the most durable responses in the table come from multi-payload constructs that couple a recruiting chemokine to a sustaining cytokine, such as CCL5 + IL-15, CCL5 + IL-12, and the triple-payload CCL5 + IL-12 + IFN-γ design, consistent with the principle that recruitment without persistence is insufficient [25,66,67,68]. Third, platform choice shapes outcome independently of payload, as the CCL19-armed vaccinia construct that improved survival without accelerating viral clearance illustrates [29]. These patterns, rather than any single construct, constitute the translatable lessons of the preclinical record, and they frame the platform and multi-payload considerations developed in Section 6 and Section 7.

## 6. The Underappreciated Role of Viral Platform Selection

**Figure 2:**
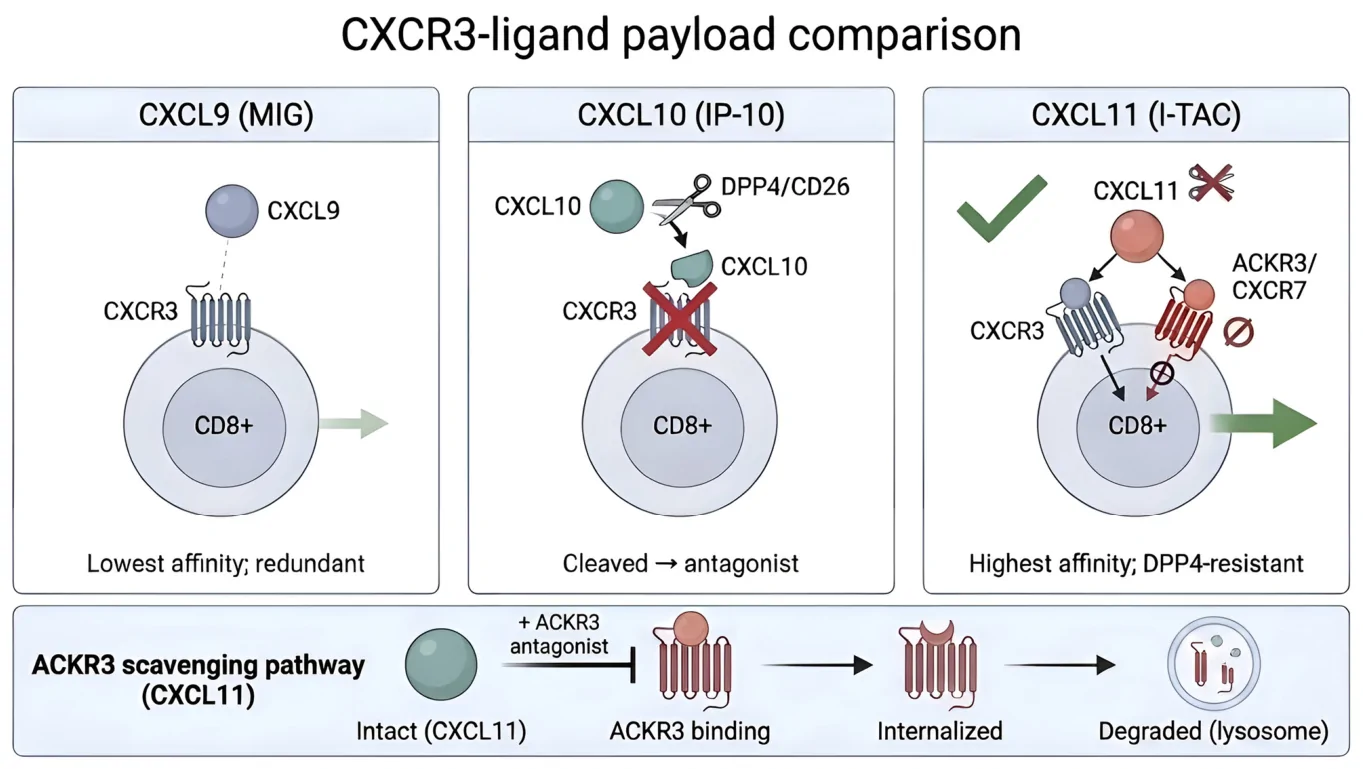
**Differential properties of the CXCR3-ligand chemokines as oncolytic-virus payloads.** The three CXCR3 ligands differ in their suitability for OV arming. CXCL9 (MIG) has the lowest CXCR3-binding affinity and is functionally redundant with the CXCL9/CXCL10 induced by viral infection once EZH2/PRC2-mediated silencing is reversed, so additional transgene yields little further effector recruitment. CXCL10 (IP-10) is rapidly cleaved at its N-terminus by DPP4/CD26 to a truncated form [CXCL10] that binds CXCR3 without signaling and acts as a receptor antagonist, blocking T-cell chemotaxis. CXCL11 (I-TAC) has the highest CXCR3 affinity and is the most resistant of the three ligands to DPP4/CD26 inactivation, making it the preferred payload for maximizing recruitment of CXCR3^+^ CD8^+^ T cells. CXCL11 is also the only CXCR3 ligand bound by the atypical receptor ACKR3/CXCR7, which internalizes and degrades it without initiating signaling; this dual-receptor binding is a scavenging liability rather than an advantage, and is the reason ACKR3 antagonism is considered a rational combination partner for CXCL11-armed constructs. The lower panel traces this scavenging route: intact CXCL11 binds ACKR3/CXCR7, is internalized, and is degraded within the lysosome without downstream signaling, progressively depleting the very gradient a CXCL11-armed virus is intended to establish. Created in BioRender. Alwithenani, A. (2026) https://BioRender.com/2epexq4 (accessed on 28 July 2026).

The therapeutic outcome of a chemokine-armed oncolytic virus depends on the interaction between payload and platform rather than on the chemokine alone (Figure 2). Three platform-intrinsic properties shape the efficacy of chemokine delivery: the endogenous chemokine profile induced by the viral backbone, replication kinetics, and transgene cargo capacity [3,13,15,16,20,22].

### 6.1. Endogenous Chemokine Baseline

Each oncolytic virus platform induces a characteristic innate immune signature on infection, and within that signature is a defined endogenous chemokine profile that sets the baseline against which any transgenic payload must compete [19,20,21,22]. Vesicular stomatitis virus elicits a strong type I interferon response with high-level CXCL9 and CXCL10 induction, which accounts for the failure of exogenous CXCL9 arming to add benefit [19]. Vaccinia virus induces moderate CXCL10 and CCL5 but only modest CXCL11, leaving substantial headroom for a CXCL11 transgene to add measurable value, a backbone–payload pairing that maps onto the comparatively consistent success of vvDD-CXCL11 constructs [22,23]. HSV-1-based platforms such as T-VEC generate their own inflammatory signature, sufficient on its own to convert the tumor microenvironment from cold to hot in a fraction of melanoma patients, which implies that the most useful payloads for this backbone are those supplying signals the virus does not already provide [1,17]. Adenoviral platforms show variable chemokine induction depending on serotype and genetic modification, which complicates prediction but creates flexibility for rational design [13,30]. The endogenous baseline therefore functions as a subtraction term: the therapeutic value of an armed chemokine is the gradient it adds beyond what the platform already produces. Awareness of these profiles is what distinguishes a rationally matched construct from a redundant one such as VSV-CXCL9 [19,69].

### 6.2. Replication Kinetics and Transgene Expression Duration

The duration and amplitude of chemokine production are governed by viral replication kinetics within the tumor [3,15,16]. Rapidly replicating viruses such as VSV produce a burst of transgene expression followed by rapid immune clearance, generating only a transient gradient [15,19]. Slower-replicating platforms such as vaccinia virus and HSV-1 sustain expression longer and, in principle, open a more prolonged window for immune cell recruitment [3,12,22]. The replication–immunity tradeoff matters particularly for chemokine-armed constructs, since effective recruitment requires gradients sustained over days to weeks rather than acute pulses [16,27,69,71].

### 6.3. Cargo Capacity and Multi-Payload Design

Practical limits on transgene insertion vary across oncolytic virus platforms [3,12,13]. Adenoviruses accommodate approximately 7.5 kb of exogenous DNA, sufficient for one to two transgene cassettes [3,13]. Vaccinia virus, with its large genome and cytoplasmic replication, tolerates insertions exceeding 25 kb, which enables multi-payload designs combining chemokines with cytokines, checkpoint antibodies, or bispecific T cell engagers (BiTEs) [3,23,72]. HSV-1 offers intermediate capacity of approximately 30 kb with multiple insertion sites [3,73,74]. Cargo capacity therefore determines the feasibility of the multi-payload strategies discussed in Section 7 [14,68,72].

## 7. Multi-Payload Strategies: Chemokines in Combination

### 7.1. Chemokine + Cytokine Dual Payloads

The dual-payload paradigm established by Nishio and colleagues with the RANTES + IL-15 construct [25,66] has been extended through progressively more elaborate designs. OncoViron (CCL5 + IL-12 + IFN-γ) is the most complex chemokine-armed oncolytic virus published to date, and it shows that triple-payload configurations can produce both local and systemic antitumor effects [68]. Fang and colleagues found that the CCL5 + IL-12 combination enhanced CAR-T cell infiltration and Stat4 phosphorylation in renal cell carcinoma, and that adding IFN-γ further potentiated inflammatory remodeling of the tumor microenvironment [67,68]. The CXCL9 + IL-15-armed adenovirus produced comparable synergy between T cell recruitment and survival in prostate cancer models [31]. The rationale for these designs is simple: antitumor immunity requires both spatial positioning (chemokines) and functional support (cytokines), and a multi-payload oncolytic virus can deliver both from a single construct [14,27,28,75].

### 7.2. OV + Immune Checkpoint Inhibitor Synergies

Combining chemokine-armed oncolytic viruses with immune checkpoint blockade addresses the principal limitation of checkpoint inhibitor therapy in cold tumors, since virus-mediated recruitment supplies the infiltrate those tumors lack [1,2,14,70,76]. Liu and colleagues found that vvDD-CXCL11 with anti-PD-L1 antibody produced synergistic tumor regression exceeding either monotherapy, the virus converting immune-excluded tumors into inflamed tumors susceptible to checkpoint blockade [70]. Clinical evidence from the T-VEC + pembrolizumab combination (the MASTERKEY-265 trial) showed a 62% overall response rate in advanced melanoma, substantially exceeding the expected efficacy of either agent alone [1]. Phase II data from T-VEC + ipilimumab likewise showed improved response rates compared with ipilimumab monotherapy [77,78]. These findings support advancing chemokine-armed oncolytic virus and checkpoint inhibitor combinations into formal clinical evaluation [16,79].

## 8. Tumor-Mediated Chemokine Antagonism

Tumors do not passively fail to recruit effector cells. They actively dismantle chemokine gradients through layered defenses at the transcriptional, post-translational, receptor, and anatomical levels, each of which can blunt the benefit of a chemokine-armed oncolytic virus [6,7,26]. At the receptor level, the atypical chemokine receptor ACKR3/CXCR7 internalizes and degrades CXCL11 and CXCL12 without initiating G-protein signaling, functioning as a molecular sink that consumes ligand and flattens the gradient required for directional migration [9]. That poses a specific tension for CXCL11-armed constructs: the ACKR3 engagement that distinguishes CXCL11 from its sister ligands is not a contributor to its activity but a separate liability, rendering the payload susceptible to ACKR3-mediated scavenging wherever the decoy receptor is overexpressed—so the payload with the highest CXCR3 affinity may also be the most rapidly cleared. At the post-translational level, DPP4/CD26 cleaves the N-terminal dipeptide of the CXCR3 ligands, converting agonists into antagonists. CXCL10 is the most vulnerable and CXCL11 comparatively resistant, a hierarchy that reinforces payload selection but applies equally to chemokine produced by an oncolytic virus, since arming raises ligand supply without exempting it from proteolysis [41,42,43,44,45]. At the transcriptional level, PRC2/EZH2-mediated H3K27 trimethylation silences the CXCL9 and CXCL10 promoters and disables the IFN-γ-driven feed-forward loop that normally amplifies effector recruitment [7,8]. An oncolytic virus supplying exogenous CXCL11 can bypass this silenced endogenous output to seed an initial gradient, but it cannot restore the amplification circuit on its own, which implies that durable recruitment may require pairing chemokine arming with epigenetic modulation. Superimposed on these molecular defenses are physical and cellular barriers: CXCL12 from FAP^+^ carcinoma-associated fibroblasts establishes a competing stromal gradient that retains T cells at the invasive margin even when an effector-recruiting chemokine is present [6,10,11,80], and tumor endothelial cells erect a final gate, expressing FasL to delete transmigrating effector T cells and endothelin B receptor to impede their extravasation [81,82]. The cumulative implication is that recruitment is necessary but not sufficient, and that the ceiling observed for single-payload chemokine arming reflects these parallel antagonistic pathways rather than any deficiency of the payload itself. Combining chemokine-armed oncolytic viruses with DPP4 inhibitors, anti-CXCL12 agents, epigenetic modulators, or ACKR3 antagonists is therefore likely to be necessary to convert a transient, locally seeded gradient into the sustained, penetrant chemoattractant field required for durable immune infiltration [6,7,41].

## 9. Translational Roadmap and Future Directions

### 9.1. Critical Evidence Gaps

Several evidence gaps must be closed before chemokine-armed oncolytic viruses can advance to rational clinical translation. First, no published study has compared different chemokine payloads head-to-head within a matched viral platform and tumor model [19,23,24,65]. The field therefore lacks the direct comparative evidence needed to rank payloads by efficacy. Second, nearly all available evidence derives from murine syngeneic or xenograft models that imperfectly recapitulate the immunosuppressive complexity of human tumors [23,25,30]. Third, the interaction between virus-delivered chemokines and pre-existing intratumoral chemokine networks remains largely uncharacterized [26,37,40]. Fourth, long-term safety data on sustained intratumoral chemokine overexpression, particularly the potential for autoimmune sequelae, are essentially absent from the preclinical literature [14,16].

### 9.2. Decision Framework by Tumor Immune Contexture

**Figure 3:**
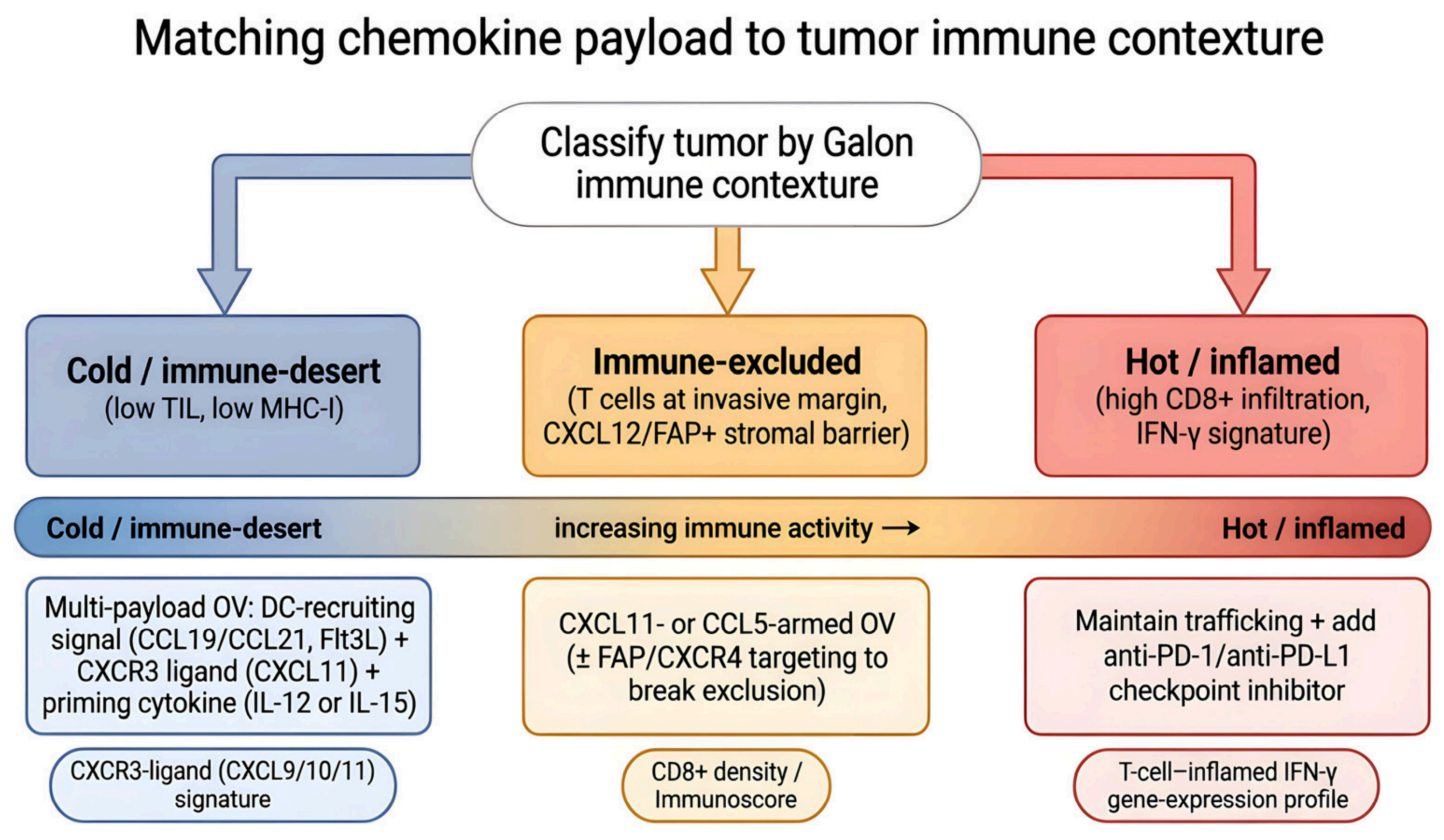
**A decision framework for matching chemokine payload to tumor immune contexture.** Tumors are classified by baseline immune contexture (Galon). Hot/inflamed tumors (high CD8^+^ infiltration, IFN-γ signature) call for maintenance of trafficking combined with anti-PD-1/anti-PD-L1 checkpoint blockade. Immune-excluded tumors (T cells confined to the invasive margin behind a CXCL12/FAP^+^ stromal barrier) are best addressed with CXCL11- or CCL5-armed OVs, optionally combined with FAP- or CXCR4-targeting to break exclusion. Cold/immune-desert tumors (low tumor-infiltrating lymphocytes, low MHC-I) require multi-payload designs that pair dendritic cell–recruiting signals (CCL19/CCL21, Flt3L) with a CXCR3 ligand (CXCL11) and a priming cytokine (IL-12 or IL-15). Recommended biomarker readouts—CXCR3-ligand (CXCL9/10/11) signature, CD8^+^ density/Immunoscore, and T-cell–inflamed IFN-γ gene-expression profile—are indicated for each branch. Created in BioRender. Alwithenani, A. (2026) https://BioRender.com/65zdwuu (accessed on 28 July 2026).

We propose that chemokine-armed oncolytic virus selection should be guided by the baseline immune contexture of the target tumor, classified according to the Galon framework into immune desert, immune excluded, and inflamed-but-suppressed phenotypes [2,5,33,34] (Figure 3). Table 2 supports this framework from the receptor side, summarizing for each axis introduced in Section 2 the ligands involved, the cells recruited, the immunological function served, the design constraints that bear on payload choice, and the viral platform best suited to delivering it. For immune desert tumors, which lack both intratumoral and peritumoral immune cells, the primary objective is de novo immune priming. CCL19-armed oncolytic viruses and dendritic cell-recruiting strategies (XCL1, CCL5) suit this objective, since they recruit antigen-presenting cells and initiate local immune responses (Table 2, CCR7 and cDC1 rows) [29,55,60,61,63]. For immune excluded tumors, in which T cells are present at the tumor margin but unable to penetrate the parenchyma, CXCL11-armed oncolytic viruses combined with anti-CXCL12 or anti-FAP strategies may overcome the stromal barrier (Table 2, CXCR3 row) [10,11,23,30,80]. For inflamed-but-suppressed tumors, in which T cells are present but functionally exhausted, the priority shifts from recruitment to reinvigoration. In this context, chemokine + checkpoint inhibitor combinations such as vvDD-CXCL11 + anti-PD-L1 [1,70,76] or multi-payload designs incorporating checkpoint antibodies [72,77,79] are the rational choices.

### 9.3. Biomarker-Guided Clinical Trial Design

Clinical translation of chemokine-armed oncolytic viruses should incorporate biomarker-driven patient selection and pharmacodynamic monitoring [34,38,39,46]. Baseline biopsies should be assessed for the Immunoscore (CD3/CD8 density at the tumor center and invasive margin) to assign immune phenotype, for intratumoral chemokine expression profiles (CXCL9, CXCL10, CXCL11, CCL5) to identify deficits amenable to virus-mediated rescue, and for antagonism markers including ACKR3/CXCR7, DPP4/CD26, and CXCL12 [9,34,37,39,41]. On-treatment biopsies should quantify changes in T cell density and clonality, chemokine gradient magnitude, and tumor microenvironment reprogramming as pharmacodynamic endpoints [1,38,46]. The IFN-γ-related mRNA signature identified by Ayers and colleagues as predictive of PD-1 blockade response is a useful baseline biomarker for identifying patients likely to benefit from chemokine-armed oncolytic virus and checkpoint inhibitor combinations [38].

### 9.4. Emerging Technologies and Future Directions

Several directions may advance the chemokine-armed oncolytic virus field. Engineering T cells to express chemokine receptors complementary to the viral payload, CXCR6-modified T cells for example, creates a bidirectional trafficking strategy that may improve the efficiency of effector cell homing [47,48]. DPP4-resistant chemokine variants, designed through N-terminal modifications that evade proteolytic cleavage, could remove a central liability of CXCR3-targeted payloads [41,44]. Synthetic biology approaches, including promoter-regulated transgene expression responsive to tumor-specific or hypoxia-inducible signals, may refine the spatiotemporal control of chemokine delivery [13,15,68]. Pairing chemokine-armed oncolytic viruses with bispecific T cell engagers (BiTEs) may address antigen heterogeneity by redirecting recruited T cells to multiple tumor targets from a single platform [27,72,75].

### 9.5. Concluding Perspective

The engineering of chemokine-armed oncolytic viruses is a rationally designed approach to one of the most persistent challenges in cancer immunotherapy: the spatial positioning of immune effector cells within the tumor microenvironment. Where checkpoint blockade releases the brakes on T cells that have already reached the tumor, and cytokine arming supports their function once there, chemokine arming addresses the prior and often rate-limiting question of whether effector cells arrive at all. The preclinical evidence reviewed here indicates that the strategy is feasible and effective, but also that its success is conditional. CXCL11 has shown the most consistent activity as a CXCR3 ligand, CCL5 is the most validated dual-payload partner, and CCL19 remains a mechanistically distinctive but underexplored option [23,25,29,30,68]. In each case the benefit depends on the interaction between payload and viral backbone, on the chemokine’s resistance to tumor-mediated antagonism, and on the immune contexture of the target lesion, rather than on the identity of the chemokine alone. The field now needs to translate the mechanistic insight accumulated from first-generation constructs into head-to-head comparative studies, biomarker-guided patient selection, and rational combinations matching chemokine–oncolytic virus pairs to defined tumor immune phenotypes [2,14,16]. As cancer immunotherapy moves toward precision combination regimens, chemokine-armed oncolytic viruses are positioned to act as the spatial architects of antitumor immunity, the agents that help determine where, and not merely whether, an antitumor response unfolds [1,14,16,27,70].

## Figures and Tables

**Table 1:** Preclinical studies of chemokine-armed oncolytic viruses.

Chemokine	OV Platform	Tumor Model	Immune Effects	Outcome	Key Insight	Ref.
CXCL9	VSV-Δ51	Murine melanoma, colon	Minimal increase over unarmed	No survival benefit	Ceiling effect: endogenous CXCL9 from VSV makes transgene redundant	[19]
CXCL11	vvDD (vaccinia)	Murine mesothelioma	Increased CD8^+^ CTLs, granzyme B	Tumor regression + systemic immunity	Unexpected abscopal vaccination effect	[23]
CXCL11	Vaccinia (VV)	Murine melanoma	Enhanced adoptive T cell infiltration	Improved ACT + vaccine efficacy	OV-delivered CXCL11 works; T cell-secreted fails	[64]
CXCL11	Oncolytic adenovirus	Murine GBM	Increased CD8^+^, NK, M1; decreased MDSC, Treg	Durable GBM control with CAR-T	TME reprogramming in immunocompetent model	[30]
CCL5 + IL-15	Ad5Δ24	Neuroblastoma xenograft	Enhanced CAR-T migration + persistence	Improved antitumor activity	Foundational “recruit + sustain” paradigm	[25,66]
CCL5	Vaccinia (vvCCL5)	Murine colon	Amplified T cell infiltration	Enhanced vaccine efficacy	CCL5 functions as immune adjuvant	[24]
CCL5 + IL-12	Ad5-ZD55	RCC xenograft	Increased CAR-T infiltration, Stat4 activation	Prolonged survival	CCL5 + IL-12 dual payload in RCC	[67]
CCL5 + IL-12 + IFN-γ	AdKi67 (OncoViron)	Multiple xenograft	CAR-T infiltration + proliferation + memory	Complete regression + abscopal	Most complex triple-payload OV construct	[68]
CXCL9 + IL-15	Adenovirus	Prostate cancer	Increased CD8^+^ T, CD45^+^CD3^+^ infiltration	Enhanced CAR-T efficacy	CXCL9 + cytokine dual-payload in prostate	[31]
CCL19	Vaccinia (vvCCL19)	Murine colon	Immune boost without viral clearance penalty	Improved survival	Unique: no accelerated viral clearance	[29]
MIP-3α	NDV	Murine melanoma	DC attraction, ICD induction	Enhanced antitumor immunity	DC-recruiting OV approach	[32]

**Table 2:** Receptor–function map for chemokine payload selection.

Receptor	Ligands	Target Cells	Function	Design Notes	Best Platform	Evidence
CXCR3	CXCL9, CXCL10, CXCL11	CD8^+^ T, Th1 CD4^+^, NK	Effector T cell homing	CXCL11 preferred: highest affinity, relatively DPP4-resistant (though also processed by CD13/APN); CXCL10 readily cleaved to a CXCR3 antagonist	VV (low endogenous CXCL11) or Ad	Strong [19,23,30,41,45,64]
CCR5	CCL3, CCL4, CCL5	T, NK, monocytes	Broad immune recruitment	CCL5 most validated; best in dual-payload with IL-15 or IL-12. Double-edged: also recruits Treg and MDSC, favoring local virally restricted delivery over sustained exposure	Ad5 (proven with CAR-T)	Strong [25,49,52,66,67,68]
CCR7	CCL19, CCL21	Naïve T, DC, central memory	TLS formation, DC positioning	CCL19 underexplored; unique immune vs. antiviral balance	VV (demonstrated)	Limited [29,53,54,55,56]
CCR2	CCL2	Monocytes, MDSC	Dual-edged: tumoricidal vs. immunosuppressive	High risk of MDSC recruitment; requires careful context	Avoid unless combined	Theoretical [57,58]
CX3CR1	CX3CL1	CTL, NK	Cytotoxic cell recruitment	Membrane-bound form may provide adhesion in addition to chemotaxis; not yet tested in an oncolytic virus context	HSV (large cargo)	Emerging [59]
CXCR3 (DC)	CXCL9, XCL1	cDC1	DC recruitment for cross-priming	Upstream of T cell infiltration; targets immune deserts	Any platform	Moderate [40,60,61,63]

## Data Availability

No new data were created or analyzed in this study. Data sharing is not applicable to this article.
